# Emergence of *Phytobacter diazotrophicus* carrying an IncA/C_2_ plasmid harboring *bla*_NDM-1_ in Tokyo, Japan

**DOI:** 10.1128/msphere.00147-23

**Published:** 2023-07-14

**Authors:** Hiroaki Kubota, Tomohiro Nakayama, Tsukasa Ariyoshi, Satomi Uehara, Yumi Uchitani, Sachio Tsuchida, Hiroyuki Nishiyama, Ichiro Morioka, Tsugumichi Koshinaga, Akiko Kusabuka, Naoki Nakatsubo, Takuya Yamagishi, Yuri Tabuchi, Rumi Okuno, Kai Kobayashi, Morika Mitobe, Keiko Yokoyama, Takayuki Shinkai, Jun Suzuki, Kenji Sadamasu

**Affiliations:** 1 Department of Microbiology, Tokyo Metropolitan Institute of Public Health, Shinjuku-ku, Tokyo, Japan; 2 Division of Laboratory Medicine and Companion Diagnostics, Department of Pathology and Microbiology, Nihon University School of Medicine, Itabashi-ku, Tokyo, Japan; 3 Clinical Laboratory Department, Surugadai Nihon University Hospital, Chiyoda-ku, Tokyo, Japan; 4 Department of Pediatrics and Child Health, Nihon University School of Medicine, Oyaguchi, Itabashi-ku, Tokyo, Japan; 5 Department of Pediatric Surgery, Nihon University School of Medicine, Itabashi-ku, Tokyo, Japan; 6 Department of Planning and Coordination, Tokyo Metropolitan Institute of Public Health, Shinjuku-ku, Tokyo, Japan; 7 Antimicrobial Resistance Research Center, National Institute of Infectious Diseases, Shinjuku-ku, Tokyo, Japan; JMI Laboratories, North Liberty, Iowa, USA

**Keywords:** Enterobacteriaceae, antibiotic resistance, plasmid-mediated resistance, molecular epidemiology, genome analysis, genotypic identification

## Abstract

*Phytobacter diazotrophicus* is an Enterobacterales species that was originally identified as a plant growth-promoting, Gram-negative bacterium. Recently, this species has been recognized as relevant to opportunistic human and nosocomial infections in clinical settings. Its frequent misidentification as other Enterobacterales species from clinical examination occasionally causes a delay in the identification of nosocomial outbreaks. Here, we report the emergence of New Delhi metallo-β-lactamase (NDM)-producing *P. diazotrophicus* isolated from hospitalized pediatric patients and hospital environments in Tokyo, Japan. In our case, these isolates were found during an investigation of carbapenem-resistant Enterobacterales in relation to nosocomial infections. Whole-genome sequencing is useful for overcoming the difficulty of species identification. Furthermore, we found that *bla*_NDM-1_ was carried by an IncA/C_2_ plasmid (approximately 170 kbp), which was transferrable from the clinical isolates to the recipient strain *Escherichia coli* J53. Our study demonstrated that *P. diazotrophicus* behaves as a carrier of *bla*_NDM_-harboring plasmids, potentially disseminating resistance to carbapenems among Enterobacterales.

**IMPORTANCE:**

Early detection of nosocomial outbreaks is important to minimize the spread of bacteria. When an outbreak is caused by multidrug-resistant bacteria such as carbapenem-resistant Enterobacterales, a delay in findings makes it difficult to control it because such bacteria often spread not only among human patients but also in hospital environments. *Phytobacter diazotrophicus*, an Enterobacterales species that has recently been found to be relevant to clinical settings, is often misidentified as other bacteria in clinical laboratories. Here, we found NDM-producing *P. diazotrophicus* in hospitalized pediatric patients and their environment in Tokyo, Japan. Given that the isolates carried *bla*_NDM-1_-harboring transferrable plasmids, the influence of such bacteria could be greater with the mediation of horizontal transfer of carbapenem resistance. Our findings suggest that *P. diazotrophicus* should be recognized as an NDM-carrier, for which more attention should be paid in clinical settings.

## OBSERVATION

Carbapenem resistance in Enterobacterales is frequently acquired through horizontal transfer of carbapenemase genes mediated by plasmids, and this transfer has occurred among different Enterobacterales species (1). The spread of a plasmid carrying carbapenemase genes among several Enterobacterales species often causes large-scale (2, 3) or small-scale (4) outbreaks. One group of the globally disseminated carbapenemase genes is the *bla*_NDM_ family, which was first identified in New Delhi, India, in 2008 (5). NDM-producing bacteria generally exhibit high levels of resistance to most β-lactams, including carbapenems, and the *bla*_NDM_ family is mostly carried by plasmids (6).

*Phytobacter diazotrophicus* was originally isolated from wild rice and first noticed as a plant-associated bacteria (7). *P. diazotrophicus* promotes plant growth via nitrogen fixation, but this species often causes opportunistic human infections, occasionally causing nosocomial outbreaks (8). *P. diazotrophicus* has frequently been misidentified as other Enterobacterales species, such as *Pantoea* spp., because of the difficulty in identification using general methods (9). As a result, a certain proportion of *P. diazotrophicus* isolates may not be precisely identified as this species, even though they are present and significant in clinical settings (10). In fact, the genus *Phytobacter* has been recognized as a new member with clinical significance (11, 12).

Here, we report the emergence of *P. diazotrophicus*, which produces an NDM-type metallo-β-lactamase. In September 2020, a NDM-producing *Klebsiella pneumoniae* was isolated from a hospitalized patient at Nihon University Itabashi Hospital in Tokyo, Japan, and NDM-producing Enterobacterales were screened in other hospitalized patients and environments where NDM-producing *K. pneumoniae* was isolated during an outbreak investigation. Consequently, several NDM-producing Enterobacterales were found; however, identifying the bacterial species for four of these, isolated from three hospitalized pediatric patients in a pediatric ward and one environmental specimen around them (Table 1), was difficult. All three patients had suffered from severe congenital disorders at the time of admission. Thus, they had received many medical practices including antibiotic treatments, which may put them in a situation where they tended to carry antimicrobial-resistant organisms. Although fever had developed on all three patients during hospitalization, this was indistinguishable from the results of chronic diseases, and no signs and symptoms of infectious diseases had been detected. Finally, the patients were handled as carriers of these bacteria after detecting NDM-producing bacteria from their nonsterile sites (feces or biliary drain) (Table 1), and contact precautions were implemented. Minimum inhibitory concentrations (MICs) were determined using a RAISUS instrument (Nissui Pharmaceutical Co., Tokyo, Japan). NDM production was examined using NG-Test CARBA 5 (NG Biotech, Guipry, France). This study was approved by the Ethics Committee of the Nihon University Itabashi Hospital (RK-210608-1). Informed consent was obtained via an opt-out form, which clarified the current study on the website of the Nihon University Itabashi Hospital (https://www.itabashi.med.nihon-u.ac.jp/cr/open_information.html).

**TABLE 1:** Isolation profiles and MICs of clinical isolates

Isolate	TA9730		TA9734		TA9759		TA9832	
Patient/environment	Patient 1		Patient 2		Environment		Patient 3	
Isolation date	2020.09.02		2020.09.04		2020.09.08		2020.09.09	
Gender	Female		Female		N/A^*c*^		Female	
Age (years)	5		5		N/A		4	
Origin	Feces		Feces		Waste channel		Biliary drain	
MIC (µg/mL)^*a*^								
Ampicillin-sulbactam	>16	R	>16	R	>16	R	>16	R
Piperacillin-tazobactam	>64	R	>64	R	>64	R	>64	R
Cefazolin	>16	R	>16	R	>16	R	>16	R
Cefotiam^*b*^	>4	−	>4	−	>4	−	>4	−
Cefotaxime	>32	R	>32	R	>32	R	>32	R
Ceftazidime	>16	R	>16	R	>16	R	>16	R
Cefepime	>16	R	>16	R	>16	R	>16	R
Cefmetazole	>32	R	>32	R	>32	R	>32	R
Moxalactam	>32	R	>32	R	>32	R	>32	R
Imipenem	>8	R	8	R	>8	R	8	R
Meropenem	>8	R	>8	R	>8	R	>8	R
Doripene	>8	R	>8	R	>8	R	>8	R
Aztreonam	≤1	S	≤1	S	≤1	S	≤1	S
Gentamicin	>8	R	>8	R	>8	R	>8	R
Tobramycin	>8	R	>8	R	>8	R	>8	R
Amikacin	>32	R	>32	R	>32	R	>32	R
Ciprofloxacin	≤0.06	S	0.5	I	≤0.06	S	0.5	I
Levofloxacin	≤0.12	S	2	R	≤0.12	S	1	I
Minocycline	≤1	S	2	S	≤1	S	2	S
Sulfamethoxazole/trimethoprim	>80	R	>80	R	>80	R	>80	R
Carbapenemase production	NDM (+)		NDM (+)		NDM (+)		NDM (+)	

^*a*^ Antibiotic susceptibility as susceptible (S), intermediate (I), or resistant (R) was determined in accordance with the MIC Breakpoints for Enterobacterales in the Clinical and Laboratory Standards Institute criteria (13).

^*b*^ There are no criteria for cefotiam (13).

^*c*^ N/A, not applicable.

The initial identification using the MALDI Biotyper system (Bruker, Billerica, MA, USA) indicated that these four isolates were possibly *Cronobacter sakazakii* or *Pluralibacter gergoviae* with low identification scores (<2.0). The ID 32 E Api Kit (bioMérieux, Marcy-l’Etoile, France) indicated that one of these was *Pantoea* spp. Due to the necessity of another approach for identification, 16S rRNA sequencing using a MicroSEQ 500 16S rDNA Sequencing Kit (Thermo Fisher Scientific, Massachusetts, CA, USA) was performed, and a BLAST search for these sequences on the NCBI website using the megablast algorithm made another candidate, *P. diazotrophicus* (Table S1).

Finally, we employed whole-genome sequencing (WGS) as a definitive analysis. A next-generation sequencing (NGS) library was prepared from genomic DNA using the Nextera XT DNA Library Preparation Kit (Illumina, San Diego, CA, USA) to obtain 2 × 300 bp paired-end short reads on the MiSeq platform (Illumina). Quality trimming was performed using Trim Galore v.0.6.7 (https://www.bioinformatics.babraham.ac.uk/projects/trim_galore) and assembled using Spades v.3.12.0 (14). The obtained contigs and reference sequences were applied to Prokka v.1.14.6 (15) for gene prediction. Core genes defined as having more than 95% identity were extracted and connected for core genome alignment using Roary v.3.13.0 (16). A distance matrix based on the average nucleotide identity (ANI) (17) was estimated using the Kostas Lab website (18). Phylogenetic trees for both methods were constructed using the MEGA7 software (19).

**Fig 1:**
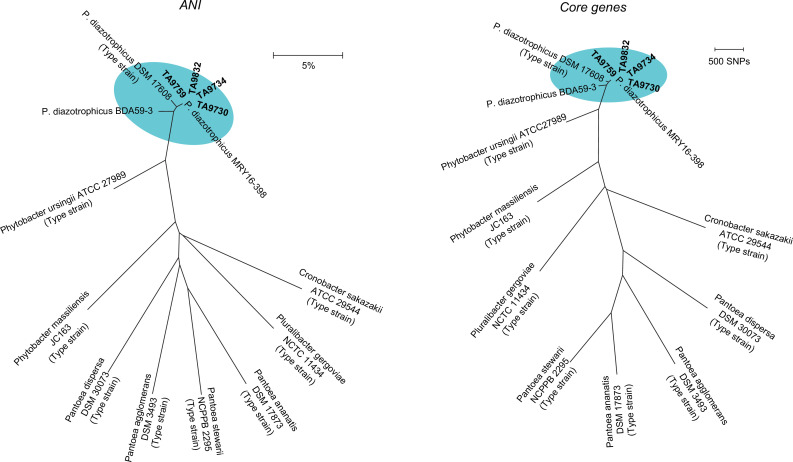
Phylogenetic trees based on whole-genome data. Clinical isolates were compared to reference strains based on ANI (left) and core genes (right). These trees were constructed using the neighbor-joining method. Clinical isolates and *P. diazotrophicus* references are shown by the elliptical area highlighted in cyan. *Metakosakonia* sp. MRY16-398 and *Citrobacter* sp. BDA59-3 are regarded as *P. diazotrophicus* MRY16-398 and *P. diazotrophicus* BDA59-3, respectively, and the reclassification of *Metakosakonia massiliensis* to *Phytobacter massiliensis* is included, according to recent studies (10, 21). Scale bars = distance.

All four isolates were much closer to *P. diazotrophicus* than other Enterobacterales species in terms of ANI and core-gene similarity (Fig. 1). The ANI values among these isolates and *P. diazotrophicus* references were less than 5%, which is considered identical (17). *Metakosakonia* sp. MRY16-398 (20), which was later identified as *P. diazotrophicus* (10, 21), was closest to our four isolates. Besides the close epidemiological relationship such as their isolations within a week (Table 1), the clonality observed in the pulsed-field gel analysis demonstrated that *P. diazotrophicus* have disseminated through nosocomial infections in the current cases (Fig. S1). After coordinated, enhanced infection control measures by the hospital and the responsible public health authorities, no additional NDM-producing Enterobacterales have been identified in the hospital.

After the current study concluded, we conducted a more thorough investigation of the isolates in April 2023. First, we tested Type Strain Genome Server, another genome-based species identification tool (22). The contig data used for ANI and core-gene analyses with all four isolates were matched with *Kluyvera intestini* GT-16 and *P. diazotrophicus* DSM 17806, with more than 80% digital DNA-DNA hybridization scores, which is sufficient for species identification (22). Given that *K. intestini* GT-16 was recently reclassified as *P. diazotrophicus* (10, 21), these findings support the original identification of our isolates as *P. diazotrophicus*. Second, we reanalyzed our isolates after the database for the MALDI Biotyper system (the MBT Compass reference library, MBT-BDAL-10833, Bruker) was updated in our lab in April 2022. As a result, *Phytobacter ursingii* was hit with all four isolates with high scores: 2.22, 2.06, 2.11, and 1.99, for TA9730, TA9734, TA9759, and TA9832, respectively. The species identification is less accurate with the MALDI system than with WGS, but it is useful for the recognition of the *P. diazotrophicus* outbreak because all four isolates are determined to be identical species.

To investigate the carriage of plasmids, long-read sequencing was performed using a MinION sequencer (Oxford Nanopore Technologies, Oxford, UK) with a library prepared using the Native Barcoding Kit (Oxford Nanopore Technologies). After quality trimming using NanoFilt v.0.1.0 (23) and adaptor trimming using Porechop v.0.2.4 (https://github.com/rrwick/Porechop), these long reads were assembled with trimmed paired-end short reads using Unicycler v.0.4.8 (24). Genes were predicted and annotated using the DFAST pipeline (https://dfast.ddbj.nig.ac.jp). Gene markers for plasmid replicon type and antimicrobial resistance (AMR)-related genes were identified using PlasmidFinder (25) and ResFinder (26), respectively.

**Fig 2:**
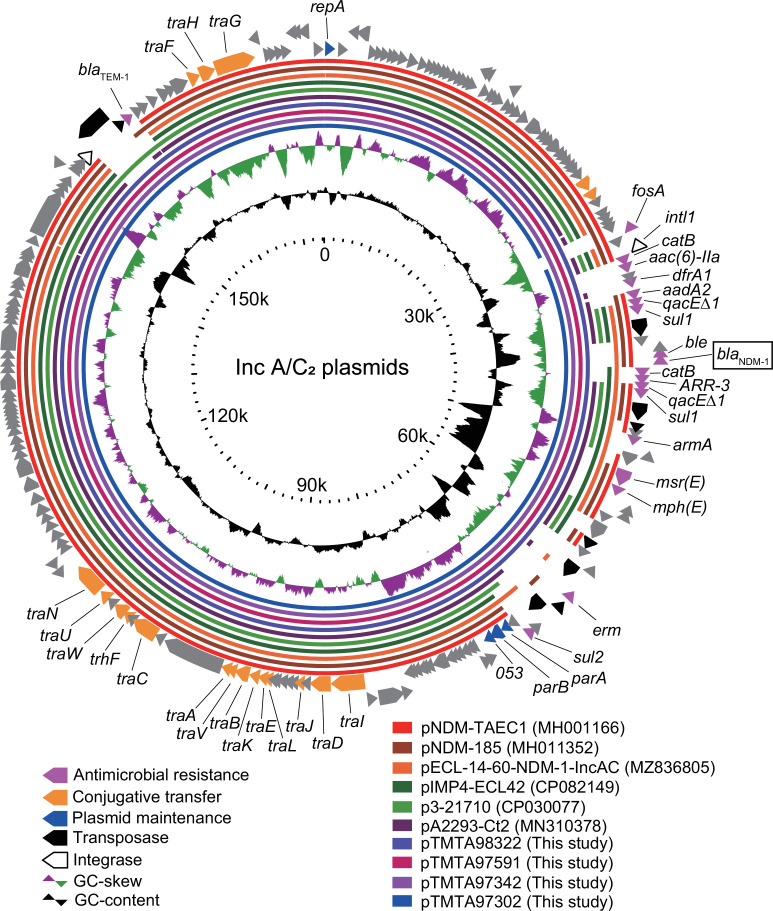
Circular map of the pTMTA97342 plasmid. IncA/C_2_ plasmids were compared and visualized using the GView Server (27). Alignment length and percentage of identity cutoff values for sequence-based BLASTn analysis revealed that the colored bars for these plasmids were 80% and 100%, respectively. The positions of open reading frames were derived from those in pTMTA98742, except for *repA*, which was derived from pNDM-TAEC1.

Four *P. diazotrophicus* isolates carried IncFIB(K) and IncA/C_2_ plasmids (Table 2), and multiple antimicrobial resistance genes, including *bla*_NDM-1_, were harbored by IncA/C_2_ plasmids. TA9734, TA9759, and TA9832 carried 174,343 bp IncA/C_2_ plasmids named pTMTA97342, pTMTA97591, and pTMTA98322, respectively. These three 174,343 bp plasmids were identical. The IncA/C_2_ plasmid from TA9730 (pTMTA97302) was identical to the other three plasmids, except for the lack of a fosfomycin-resistant gene (*fosA*) (Table 2; Fig. 2), where a slight modification of the plasmid occurred on pTMTA97302 during a series of nosocomial infections. pTMTA97342 shared nucleotide sequences of *bla*_NDM_-harboring IncA/C_2_ plasmids pECL-14–60-NDM-1-IncAC, pNDM-185, and pNDM-TAEC1 with 90%, 89%, and 89% identity, respectively. In addition, it was closely related to *bla*_NDM_-non-harboring IncA/C_2_ plasmids pA2293-Ct2, p3-20710, and pIMP4-ECL42 (with 92%, 90%, and 90% identities, respectively), suggesting that the common backbone of these plasmids contributes to the dissemination among Enterobacterales, even though the β-lactamase genes carried are diverse.

**TABLE 2:** Genetic profile of *P. diazotrophicus* isolates

Isolate	Chromosome/plasmids^*b*^	Accession no.	Length (bp)	Plasmid replicon type	AMR-related genes
TA9730	Chromosome	AP028041	5,610,297	N/A^*a*^	Not found
	pTMTA97301	AP028042	175,916	IncFIB(K)	Not found
	**pTMTA97302**	**AP028043**	**172,011**	**IncA/C**_**2**_	***ARR-3*****,** ***aac(6')-IIa*****,** ***aadA2*****,** ***armA*****,** ***bla***_**NDM-1**_ **,** ***bla***_**TEM-1B**_ **,** ***catB4*****,** ***dfrA1*****,** ***mph(E)*****,** ***m*** **sr(E),** ***sul1*****,** ***sul2***
	pTMTA97303	AP028044	98,997	Not identified	Not found
	pTMTA97304	AP028045	3,530	Not identified	Not found
	pTMTA97305	AP028046	2,496	Not identified	Not found
TA9734	Chromosome	AP025334	5,709,362	N/A	Not found
	pTMTA97341	AP025335	174,856	IncFIB(K)	Not found
	**pTMTA97342**	AP025336	**174,343**	**IncA/C**_**2**_	***ARR-3*****,** ***aac(6')-IIa*****,** ***aadA2*****,** ***armA*****,** ***bla***_**NDM-1**_ **,** ***bla***_**TEM-1B**_ **,** ***catB4*****,** ***dfrA1*****,** ***fosA3*****,** ***mph(E)*****,** ***msr(E)*****,** ***sul1*****,** ***sul2***
	pTMTA97343	AP025337	3,530	Not identified	Not found
	pTMTA97344	AP025338	2,496	Not identified	Not found
TA9759	Chromosome	AP028047	5,709,306	N/A	Not found
	**pTMTA97591**	**AP028048**	**174,343**	**IncA/C**_**2**_	***ARR-3*****,** ***aac(6')-IIa*****,** ***aadA2*****,** ***armA*****,** ***bla***_**NDM-1**_ **,** ***bla***_**TEM-1B**_ **,** ***catB4*****,** ***dfrA1*****,** ***fosA3*****,** ***mph(E)*****,** ***msr(E)*****,** ***sul1*****,** ***sul2***
	pTMTA97592	AP028049	173,698	IncFIB(K)	Not found
TA9832	Chromosome	AP028050	5,675,557	N/A	Not found
	pTMTA98321	AP028051	174,856	IncFIB(K)	Not found
	**pTMTA98322**	**AP028052**	**174,343**	**IncA/C**_**2**_	***ARR-3*****,** ***aac(6')-IIa*****,** ***aadA2*****,** ***armA*****,** ***bla***_**NDM-1**_ **,** ***bla***_**TEM-1B**_ **,** ***catB4*****,** ***dfrA1*****,** ***fosA3*****,** ***mph(E)*****,** ***msr(E)*****,** ***sul1*****,** ***sul2***
	pTMTA98323	AP028053	3,530	Not identified	Not found
	pTMTA98324	AP028054	2,496	Not identified	Not found

^*a*^ N/A, not applicable.

^*b*^ IncA/C_2_ plasmids harboring *bla*_NDM-1_ are shown in bold characters.

The conjugative transfer of pTMTA97302 and pTMTA97342 from clinical isolates to *Escherichia coli* J53 was tested using the mating method (28). The transfer frequency, calculated according to a previous report (29), was approximately 2.8 × 10^−3^ and 4.1 × 10^−3^ for pTMTA97302 and pTMTA97342, respectively.

In summary, our initial identification using general methods had failed to identify *P. diazotrophicus* as shown by the previous reports which mentioned frequent misidentification of this species (9, 10), and the difficulty in identification was overcome using whole-genome analysis. Even though *P. diazotrophicus* is an opportunistic pathogen (10), the correct identification is important to prevent delays in detecting nosocomial outbreaks. In fact, multi-state sepsis outbreaks caused by contaminated total parenteral nutrition had been reported in Brazil (9). Notably, such a delay can be more serious because this species often carries antimicrobial resistance genes including *bla*_KPC_ and *bla*_IMP-6_ (10). Our study consolidates the importance of focusing on *P. diazotrophicus* because our isolates carried a *bla*_NDM-1_-harboring plasmid, which could spread carbapenem resistance via the horizontal transfer of plasmids in clinical settings. Considering the potential as a carrier of antimicrobial resistance genes, *P. diazotrophicus* should be more recognized as a clinically relevant pathogen.

## Data Availability

The raw sequence reads and the complete genome sequences in this study were deposited in the DNA Data Bank of Japan, under the BioProject ID PRJDB12598.

## SUPPLEMENTAL MATERIAL

Table S1, Figure S1, and supplemental references msphere.00147-23-s0001.pdf BLAST hit results on partial 16S rRNA sequence and pulsed-field gel electrophoresis for four *P. diazotrophicus* isolates.

ASM does not own the copyrights to Supplemental Material that may be linked to, or accessed through, an article. The authors have granted ASM a non-exclusive, world-wide license to publish the Supplemental Material files. Please contact the corresponding author directly for reuse.
