# Supplementary material for: Emergence of Phytobacter diazotrophicus carrying an IncA/C2 plasmid harboring blaNDM-1 in Tokyo, Japan
Source: mSphere. 2023 Jul 14;8(4):e00147-23. doi: 10.1128/msphere.00147-23 (PMC10449528; doi:10.1128/msphere.00147-23)
Supplement: Table S1, Figure S1, and supplemental references — BLAST hit results on partial 16S rRNA sequence and pulsed-field gel electrophoresis for four P. diazotrophicus isolates. [file msphere.00147-23-s0001.pdf]

**Table S1. BLAST hit results on partial 16S rRNA sequence. ***

| Identity % | Species | Strain | Identical bases | Accession No. |
|---|---|---|---|---|
| 99.59 | *Phytobacter diazotrophicus* | UAEU22 | 484/486 | CP051548 |
| | *Metakosakonia* sp. | MRY16-398 | 484/486 | AP018756 |
| | *Phytobacter* sp. | SCO41** | 484/486 | CP027225 |
| | *Phytobacter diazotrophicus* | DSM 17806 | 484/486 | KY288669 |
| 99.38 | *Phytobacter* sp. | SCO41** | 483/486 | KT192640 |
| | *Citrobacter* sp. | BDA59-3 | 483/486 | CP063427 |
| | *Grimontella senegalensis* | C1p | 483/486 | AY217653 |
| | *Yokenella* sp. | UR 6-12 | 480/483 | KM253221 |
| 99.18 | *Phytobacter diazotrophicus* | BASG96 | 483/487 | MN647003 |
| 98.98 | *Phytobacter diazotrophicus* | 10289RM | 483/488 | KY288670 |
| | *Enterobacter* sp. | 2357 | 483/488 | JX174234 |
| 98.97 | *Phytobacter diazotrophicus* | BLM01 | 481/486 | MK894154 |

*BLAST search using the megablast algorithm was performed in 2020, and the results remained the same on March 1, 2023. Although the species of several strains such as *Metakosakonia* sp. MRY16-398 and *Citrobacter* sp. BDA59-3 were corrected later (1,2), those displayed in the NCBI-BLAST site are shown .

** Two *Phytobacter* sp. SCO14 sequences exist in the database, and both were hit.

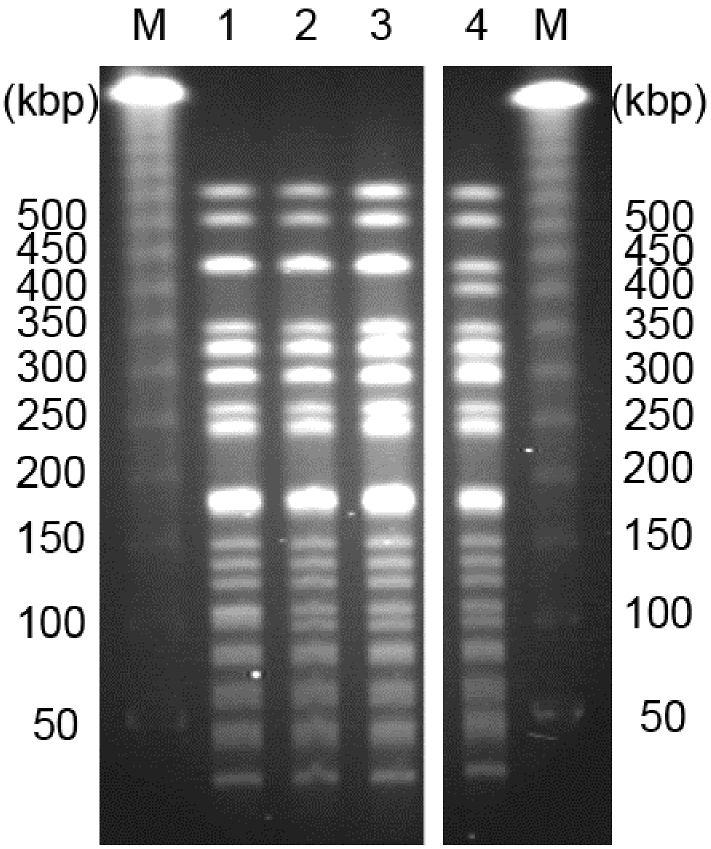


**Figure S1. Pulsed-field gel electrophoresis for four *P. diazotrophicus* isolates.**

DNA-plugs were digested by *Xba*I (New England Biolabs, Ipswich, MA, USA) and applied to electrophoresis using the CHEF mapper system (Bio-Rad, Hercules, CA, USA). In accordance with the Tenover criteria (3), these four isolates (1: TA9730, 2: TA9734, 3: TA9759, 4: TA9832) are indistinguishable. M represents a lambda DNA ladder marker (Promega, Madison, WI, USA)

**Supplementary References**


1. Smits THM, Arend LNVS, Cardew S, Tang-Hallbäck E, Mira MT, Moore ERB, Sampaio JLM, Rezzonico F, Pillonetto M. 2022. Resolving taxonomic confusion: establishing the genus *Phytobacter* on the list of clinically relevant Enterobacteriaceae. Eur J Clin Microbiol Infect Dis 41:547–558.
2. Ma Y, Yao R, Li Y, Wu X, Li S, An Q. 2020. Proposal for unification of the genus *Metakosakonia* and the genus *Phytobacter* to a single genus *Phytobacter* and reclassification of *Metakosakonia massiliensis* as *Phytobacter massiliensis* comb. nov. Curr Microbiol 77:1945–1954.
3. Tenover FC, Arbeit RD, Goering RV, Mickelsen PA, Murray BE, Persing DH, Swaminathan B. 1995. Interpreting chromosomal DNA restriction patterns produced by pulsed-field gel electrophoresis: criteria for bacterial strain typing. J Clin Microbiol. 33:2233-2239.
